# Supplementary material for: Influence of Production Factors on Beef Primal Tissue Composition
Source: Foods. 2022 Feb 11;11(4):518. doi: 10.3390/foods11040518 (PMC8871317; doi:10.3390/foods11040518)
Supplement: Supplementary file 1 [file foods-11-00518-s001.zip › foods-1557787-supplementary.pdf]

Table S1. Distribution and breed covariate grouping used in individual models of each response variable.

	Type of Variable	Distribution	Covariate Grouping
**Commercial weight**	Continuous	Gamma	AN, SM
**REA**	Continuous	Gamma	AN, SM, CH
**Grade fat**	Continuous	Gamma	AN, SM, CH
**Marbling**	Continuous	Gamma	AN, SM, CH
**Muscle score**	Categorical	Binomial	AN
**Fat class**	Categorical	Binomial	AN, SM, CH
**Dressing percentage**	Percentage	Beta	AN, SM, HH
**Estimated yield**	Percentage	Beta	AN, SM, CH
**Fat (IMF)**	Percentage	Beta	AN, SM
**Primal Cuts**			
**Round**	Percentage	Beta	AN, SM
**Loin**	Percentage	Beta	AN
**Flank**	Percentage	Beta	AN, SM, CH
**Chuck**	Percentage	Beta	none
**Rib**	Percentage	Beta	AN
**Plate**	Percentage	Beta	AN
**Brisket**	Percentage	Beta	AN
**Shank**	Percentage	Beta	AN
**Lean Component**			
**Round**	Percentage	Beta	AN, SM, CH
**Loin**	Percentage	Beta	AN, SM, CH
**Flank**	Percentage	Beta	AN, SM, CH
**Chuck**	Percentage	Beta	AN, SM, CH
**Rib**	Percentage	Beta	AN, SM, CH
**Plate**	Percentage	Beta	AN, SM, CH
**Brisket**	Percentage	Beta	AN, SM, CH
**Shank**	Percentage	Beta	AN, SM, CH
**Total**	Percentage	Beta	AN, SM, CH
**Fat Component**			
**Round**	Percentage	Beta	AN, SM, CH
**Loin**	Percentage	Beta	AN, SM, CH
**Flank**	Percentage	Beta	AN, SM, CH
**Chuck**	Percentage	Beta	AN, SM, CH
**Rib**	Percentage	Beta	AN, SM, CH
**Plate**	Percentage	Beta	AN, SM, CH
**Brisket**	Percentage	Beta	AN, SM, CH
**Shank**	Percentage	Beta	AN, SM, CH
**Total**	Percentage	Beta	AN, SM, CH

REA, ribeye area; IMF, IntAN = Angus; SM, Simmental; CH, Charolais.
